# Analysis of Pyroptosis-Related Signature for Predicting Prognosis and Tumor Immune Microenvironment in Pancreatic Cancer

**DOI:** 10.3389/fonc.2022.770005

**Published:** 2022-05-31

**Authors:** Lincheng Li, Zhaoda Deng, Zhaohui Xiao, Wenbo Zou, Rong Liu

**Affiliations:** ^1^ Medical School of Chinese PLA, Beijing, China; ^2^ Faculty of Hepato-Pancreato-Biliary Surgery, The First Medical Center, Chinese PLA General Hospital, Beijing, China

**Keywords:** pyroptosis, prognosis, immune microenvironment, pancreatic cancer, therapy

## Abstract

Pancreatic cancer (PC) has a poor prognosis, which is attributable to its high aggressiveness and lack of effective therapies. Although immunotherapy has been used for the treatment of various tumor, its efficacy in pancreatic cancer is not satisfactory. As a caspase-1-dependent programmed cell death, pyroptosis s involved in the pathological process of many tumors. Nevertheless, the vital role of the pyroptosis-related gene (PRG) in PC remains unknown. In this study, univariate COX regression was performed for 33 pyroptosis-related genes. Based on these prognosis-related PRGs, all PC patients in the Cancer Genome Atlas (TCGA) database were divided into four subtypes. Then, pyroptosis score (PP-score) was established to quantify pyroptosis level for individual PC patients using principal component analysis (PCA) algorithms. Assessment of pyroptosis level within individual PC patients may predict tumor classification and patient prognosis. Finally, a signature was constructed in TCGA and verified in ICGC. In addition, immunocheckpoint analysis revealed the possibility that the low-risk group would benefit more from immunocheckpoint therapy. Taken together, pyroptosis-related genes play a significant role in tumor immunotherapy and can be utilized to predict the prognosis of PC patients.

## Introduction

Pancreatic cancer (PC) is one of the most malignant tumors of the digestive system, which is known as the “king of cancer” (1). Pancreatic cancer, though accounting for only 3% of all cancers, is the third most deadly because of its high mortality rate (2). Despite advances in chemotherapy, radiation, and immunotherapy, pancreatic cancer still has a high mortality rate due to insensitivity to treatment (3). Therefore, early diagnosis of pancreatic cancer is an important factor affecting the survival of PC patients, which is particularly important. Tumor markers have been widely applied in the early diagnosis of cancer (4, 5). Thus, exploring and developing effective biomarkers is an important way to solve this problem.

Pyroptosis was confirmed as a new programmed cell death (PCD), which was accompanied by inflammation. With cell swelling until the cell membrane ruptures, the contents of the cell are released, and a strong inflammatory response is activated (6, 7). Pyroptosis is an important immune response in the body, which play a significant role in resistance to infection (8). The pathogenesis of pyroptosis depends on the inflammatory caspase and GSDMs protein family (9, 10). Many studies have indicated that pyroptosis may be a double-edged sword in the development and treatment of tumors, which may depend on different histological and genetic backgrounds. As an inflammatory death, the formation of inflammatory microenvironment is beneficial for the growth and invasion of tumor cells. In contrast, pyroptosis has become a new way of tumor cell death, which can inhibit the growth and spread of tumor (11). In addition, studies have shown that a variety of inflammatory pathways involved in pyroptosis may also be involved in chemotherapy and immunotherapy in cancer (12–14). It can be seen that the regulation of tumor pyroptosis may be an important approach to tumor therapy or improve the efficacy of chemotherapy. However, there are relatively few research on pyroptosis in pancreatic cancer, especially on the prognostic role of molecules related to pyroptosis.

In our research, we used consensus clustering to distinguish PC patients in TCGA into four subtypes based on prognosis-related PRGs. Next, we constructed pyroptosis score (PP-score) to evaluate the pyroptosis level of individual PC patients. Moreover, we developed a prognostic risk model that used pyroptosis-related genes to reflect the pyroptosis risk. Altogether, we investigate the prognostic value of pyroptosis-related genes and a significant role of pyroptosis in tumor immune microenvironment.

## Methods

### Data Acquisition and Performance

RNA sequencing data (FPKM value) for 177 PAAD patients and corresponding clinical data were obtained from the TCGA database (https://www.cancer.gov/tcga), which was used as a training cohort. While the validation cohorts including 288 PDAC patients from E-MTAB-6134 and 81 PDAC patients from ICGC (PACA-AU) were downloaded from the ICGC database (https://www.icgc-argo.org) and ArrayExpress (https://www.ebi.ac.uk/arrayexpress), respectively. Normalized data were log2-transformed for further analysis. In addition, PAAD somatic mutation data were acquired from TCGA. Data analysis was carried out using the R (version 4.1.1) and R Bioconductor packages. Finally, we retrieved 33 pyroptosis-related genes (PRGs) from prior literature for further analysis (15, 16).

### Consensus Clustering

Univariate Cox regression analysis was performed to determine prognostic PRGs with criteria of p <0.05. Consensus clustering was used to identify distinct patterns associated with pyroptosis using the k-means algorithm on account of prognostic PRGs. The ConsensuClusterPlus R package was performed to determine the number of clusters. Performed 1,000 iterations to ensure classification stability.

### Construction of Pyroptosis Score

In order to quantify the pyrotosis level of individual PC patient, an objective scoring system was constructed—pyroptosis score (PP-score). Principal component analysis (PCA) was performed to build a signature associated with pyroptosis. The signature scores is composed of both PC1 and PC2. This approach has the advantage of using the most relevant gene sets to construct the score, while the proportion of genes that are not associated with the major gene sets is reduced. Then use the following formula to define PP-score:


$$\text{PP}-\text{score}=\sum \left(PC1_{i}+PC2_{i}\right)$$

where *i* represents the expression of prognosis-related PRGs.

### Developing and Validating of a PRG Prognostic Model

Lasso-penalized Cox regression analysis was performed to further reduce the number of PRGs with best predictive performance using 10-fold cross validation based on glmnet package in R. A prognostic gene signature was constructed based on a linear combination of the regression coefficients (β) derived from the Lasso Cox regression model multiplied with its mRNA expression level. The formula was as follows:


$$\text{risk score}=\sum_{i=1}^{N}\left(Exp_{i}\times Coe_{i}\right)$$

Where Coei represents the corresponding PRGs coefficient, and Expi represents the corresponding PRGs expression level.

The patients were divided into low and high risk groups depending on the median risk score. Kaplan-Meier (KM) survival curve was performed using the Survminer package. In addition, receiver operating characteristic (ROC) curve was plotted to estimate the performance of the prognostic model using the “survivalROC” R package.

### Independent Prognosis Analysis and Nomogram Construction

To assess the relationship of survival prognosis with clinicopathological factors and risk score, we submitted age, gender, tumor histological grading and TNM staging for univariate and multivariate Cox regression analyses with “survival”, ”survminer” package. If a factor meets p < 0.05 in multivariate regression analysis, it is considered an independent prognostic factor. Furthermore, a nomogram to predict patients’ prognosis was established and visualized for clinicopathological factors and risk score. The discrimination performance of the nomogram was quantitatively assessed by the ROC curve. The calibration curve was drawn to validate the prediction ability of the nomogram. The closer the calibration curve was to the diagonal, the better the predictive ability of the model was.

### Gene Expression Profiling Interactive Analysis

Gene Expression Profile Analysis (GEPIA) is a comprehensive demonstration and interactive analysis website that is used to compare the expression of signature PRGs in prognostic model.

### Validation of Prognostic PRGs Using qRT‐PCR

The human pancreatic ductal epithelium cell line HPDE6-C7 and pancreatic cancer cell line BxPc-3 were purchased from the American Type Culture Collection (ATCC, Manassas, USA). Cells are cultured in high glucose DMEM (Gibco), added with 10% fetal serum bovine (Gibco), and placed in a 37°C, 5% CO2 incubator. The PC tumors and normal tissues were obtained from the Chinese PLA general Hospital. The diagnosis of PC is based on pathological examination. Perform qRT-PCR to check gene expression changes in prognostic PRGs. In short, use TRIzol reagent (Ambion) to isolate whole RNA from cancer and normal tissues, and use Eppendorf Mastercycler^®^ to convert it into cDNA according to the instructions provided. StepOnePlus Real-Time PCR System was used to perform qPCR using the primers listed in **Table 1**.

**Table 1:** Primers used for qRT‐PCR analysis.

Gene	Direction	equences (5′–3′)
18s	Forward	AACCCGTTGAACCCCATT
18s	Reverse	CCATCCAATCGGTAGTAGCG
CASP4	Forward	TCTGCGGAACTGTGCATGATG
CASP4	Reverse	TGTGTGATGAAGATAGAGCCCAT
NLRP1	Forward	GCCTTCTGTGAGAGAGAGCCT
NLRP1	Reverse	TGCAGTATGACTATGCGAGGTT
PLCG1	Forward	GGAAGACCTCACGGGACTTTG
PLCG1	Reverse	GCGTTTTCAGGCGAAATTCCA
GSDMC	Forward	TCCATGTTGGAACGCATTAGC
GSDMC	Reverse	CAAACTGACGTAATTTGGTGGC
IL-18	Forward	TCTTCATTGACCAAGGAAATCGG
IL-18	Reverse	TCCGGGGTGCATTATCTCTAC
NLRP2	Forward	TGGCCTGGAGATAGCAAAGAG
NLRP2	Reverse	CACCACCGTGTATGAGAAGGG
CASP1	Forward	TTTCCGCAAGGTTCGATTTTCA
CASP1	Reverse	GGCATCTGCGCTCTACCATC

### Statistical Analysis

The mutation landscape in patients with high and low PP-score subtype was presented with “maftools” R package in TCGA-PAAD cohort. Landscape of genomic copy number variation in chromosomes was plotted using “RCircos” R package. P <0.05 is considered statistically significant.

## Result

### Copy Number Variations of PRGs in PAAD

**Figure 1:**
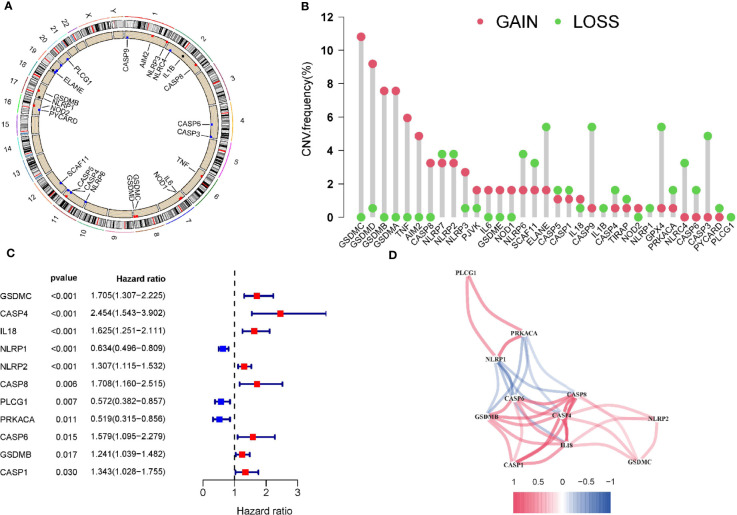
Copy number variations of PRGs in PAAD. **(A)** The location of CNV change of 33 PRG on chromosomes in the PAAD cohort. **(B)** The CNV variation frequency of 33 PRG in the PAAD cohort. **(C)**The univariate Cox regression analysis for PRGs in pancreatic cancer. **(D)** The correlation network of the pyroptosis-related genes. Red represents a positive correlation, while blue represents a negative correlation. The deeper the color, the stronger the relevance.

The expression and clinical information of 33 PRGs were obtained from TCGA. **Figure 1A** shows the location of CNV changes on the chromosome of these 33 PRGs. We also analysed the frequency of CNV changes, which indicted that there were pervasive CNV changes in these 33 PRGs. We found that the CNV amplification frequencies of AIM2, CASP8, GSDMA, GSDMB, GSDMC, GSDMD, GSDME, IL18, IL6, NLRP3, NOD1, NOD2, PJVK, TNF were widespread (**Figure 1B**). The analysis of univariate Cox regression was executed to screen the prognosis-related PRGs. The result showed that 11 PRGs were significantly related to the patient’s prognosis (p <0.05) (**Figure 1C**). The correlation network containing prognostic PRGs is presented in **Figure 1D**. Further, Gene ontology (GO) enrichment analysis and Kyoto Encyclopaedia of Genes and Genomes (KEGG) pathway analysis were then performed based on these prognostic PRGs.The results indicated that these PRGs were mainly involved in immune response, NOD-like receptor signaling pathway, and apoptotic process (**Supplementary Figure 1**).

### Tumour Classification Based on the Prognostic PRGs

**Figure 2:**
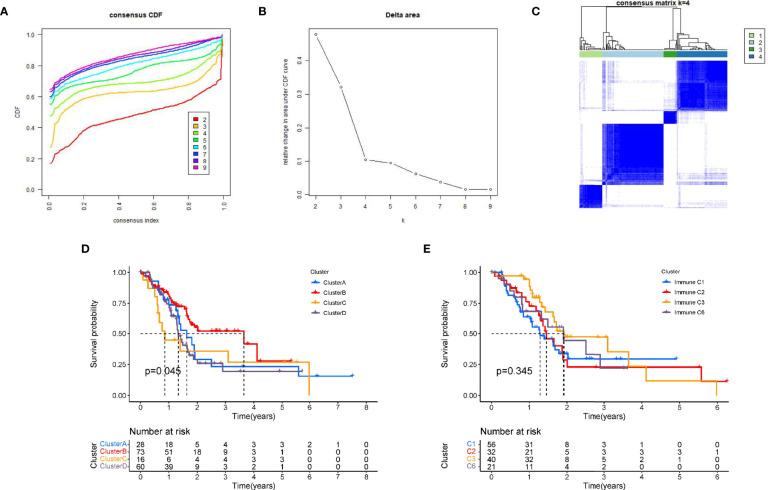
Tumour classification based on the prognostic PRGs. **(A)** The relative change of the area under the CDF curve from 2 to 10 of k. **(B)**The consistency clustering cumulative distribution function (CDF) when k is between 2 and 10. **(C)** At k=4, the correlation between groups. **(D)** Kaplan-Meier survival analysis among four clusters. **(E)** Kaplan-Meier survival analysis among immune subtypes reported in prior literature.

Further, we performed a consistent cluster analysis in the TCGA cohort based on prognostic PRGs. According to the cumulative distribution curve and the area under the distribution curve, we selected k = 4 as the optimal cluster number (**Figures 2A–C**). In addition, we validated the molecular subtypes in E-MTAB-6134, which showed that k=4 is indeed the best number (**Supplementary Figures 2A–C**). Therefore, PC patients could be well classified into four clusters according to prognostic PRGs. We analyzed the prognostic relationship between the four groups and found that the survival rate of cluster B was higher than that of other clusters (p<0.05) (**Figure 2D**). In addition, we compared above molecular subtypes with previously reprorted subtypes, in which 33 different cancers from TCGA are divided into six immune subtypes (17). However, the survival analysis curve revealed that there was no difference in four immune subtypes in PAAD (**Figure 2E**). Gene expression profiles of prognostic PRGs and clinical traits between the four clusters are shown in a heatmap (**Supplementary Figure 3**).

### Construction of Pyroptosis Score and Correlation With Tumour Classification

**Figure 3:**
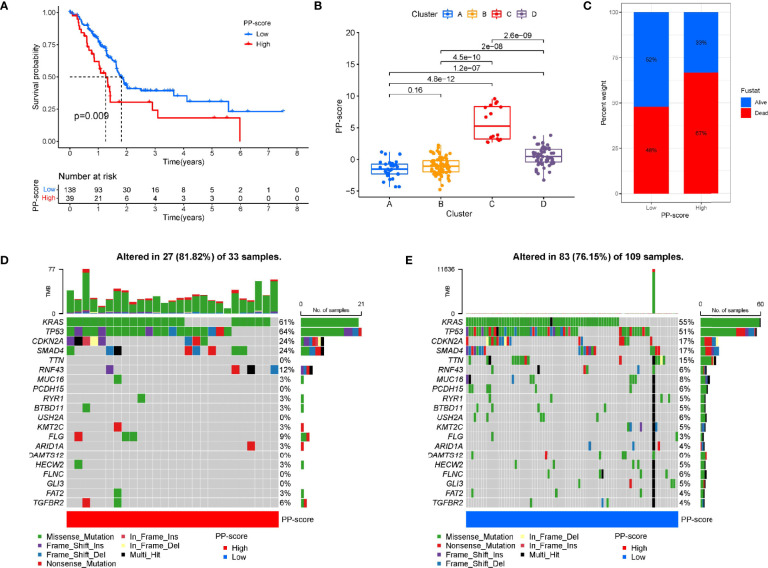
Construction of pyroptosis score and correlation with tumour classification. **(A)** Kaplan-Meier survival curve between high and low PP-score group. **(B)** The correlation between tumour classification and PP-score. **(C)**The correlation between PP-score and survival state in PAAD patients. **(D-E)** The waterfall plot of tumor somatic mutation in high PP-score and low PP-score group.

Based on these prognosis-related PRGs, a set of scoring system was established to quantify pyroptosis level for individual PC patients. This is called the pyrotosis score (PP score). Next, PC patients were divided into low or high PP-score group using the cutoff value determined by “survminer” R package. PC patients with low PP scores showed significant life-prolonging effects (**Figure 3A**). To investigate the association between PC subtypes and PP-score, we analyzed differences in PP-score between above PC subtypes. Except that there was no difference between A and B, there were significant differences in four subtypes, and the median score in cluster C was highest (**Figure 3B**). Then we compared the relationship between PP-scores and patient survival state. We found that more PC patients were dead in the high-scoring group than in the low-scoring group (**Figure 3C**). Next, the maftools package was used to analyze the difference in somatic mutation distribution between low and high PP-score in TCGA-PAAD cohort. As shown in **Figures 3D–E**, high PP-score group presented more extensive tumor mutation burden than the low PP-score group (81.82% vs 76.15%).

### Construction of a Pyroptosis-Related Prognostic Model

**Figure 4:**
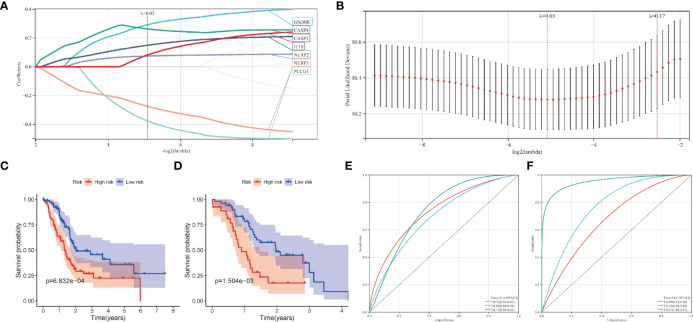
Construction of the pyroptosis-related prognostic model in pancreatic cancer. **(A, B)** The lasso-penalized Cox regression analysis for pyroptosis-related genes in TCGA cohort. **(C, D)** Kaplan-Meier survival analysis of high-risk and low-risk group in TCGA and ICGC cohort. **(E, F)** Receiver operating characteristic (ROC) curves for 1, 2, and 3 years survival in TCGA and ICGC cohort.

Firstly, after excluding patients with 0-month survival, a predictive signature was establish using lasso-penalized Cox regression analysis for PC patients (**Figures 4A–B**). The risk score of each PC sample was calculated as follows: risk score = (0.292269 × Exp_GSDMC_) + (0.263447 × Exp_CASP4_) + (0.149511 × Exp_IL18_) + (-0.274430 × Exp_NLRP1_) + (0.07476 × Exp_NLRP2_) + (-0.376026 × Exp_PLCG1_) + (0.081800 × Exp_CASP1_).

**Figure 5:**
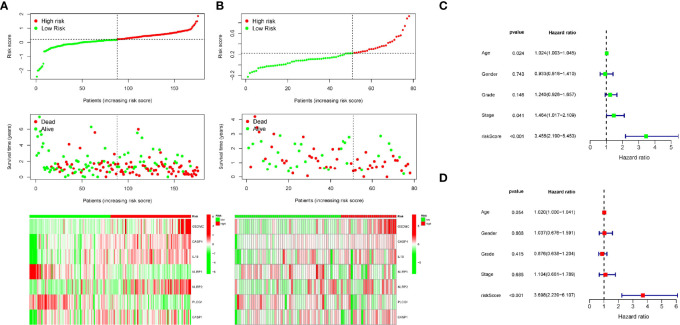
Univariate and multivariate Cox regression analysis of clinical features and the signature. **(A, B)** The distribution of risk score (upper), survival time (middle) and gene expression of PRGs (below) in TCGA and ICGC cohort. **(C)** Univariate Cox regression analysis for the prognostic signature. **(D)** Multivariate Cox regression analysis for the prognostic signature.

Patients in the training cohort were then divided into high- and low-risk groups according to the median risk level. In particular, the survival curve revealed that PC patients in the low-risk group have significantly longer survival times than the high-risk group (**Figure 4C**). Then we calculated the AUC value to evaluate the sensitivity and specificity of the risk score for predicting prognosis of PC patients. As shown in **Figure 4E**, the AUC at 1-, 2-, and 3- years was 0.73, 0.69, and 0.77 respectively, suggesting that had a high predictive value for the prognosis of PC patients. **Figure 5A** shows the distribution of prognostic model, the survival results of different groups of PC patients, and the expression profiles of selected PRGs.

Moreover, we also analyzed the predictive values of the prognostic model in the validation cohort (PACA-AU). Consistent with the results of the training cohort, the survival curve in the validation cohort showed that PC patients in the low-risk group had better survival outcomes than in the high-risk group (**Figure 4D**). The AUC at 1, 2, and 3 years were 0.69, 0.78, 0.97 in the validation cohort (**Figure 4F**).The distribution of risk score, survival state and expression profiles of PRG were shown in **Figure 5B**.

### Building a Predictive Nomogram

**Figure 6:**
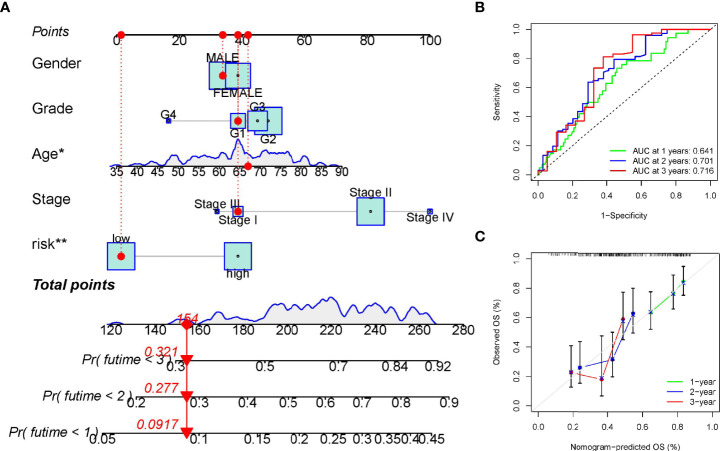
Establishment and verification of the overall survival nomogram for pancreatic cancer patients. **(A)** Construction of a nomogram with risk score and clinical characteristics. **(B, C)** Receiver operating characteristic (ROC) and calibration curve for verifying the accuracy of nomogram. *p < 0.05; **p < 0.01.

The above analysis has shown that this signature works well. Next, to investigate whether the prognostic signature was independent of other clinicopathological factors, univariate and multivariate Cox regression analysis were performed. Results showed that the signature was indeed an independent prognostic factors for PC patients (**Figures 5C–D**). In addition, a nomogram consisting of risk score and clinical features was designed to predict a survival rate of 1/2/3 years (**Figure 6A**). The AUC at 1, 2, and 3 years were 0.641, 0.701, 0.716, respectively (**Figure 6B**). Further, the calibration curve showed that the predicted overall survival rate of 1/2/3 years was well in line with the actual observation results (**Figure 6C**). All the above results confirmed that the nomogram model has obvious reliability in judging the prognosis of PC patients.

### The Relationship of the Prognostic Signature and Immune Cell Infiltration in PAAD

**Figure 7:**
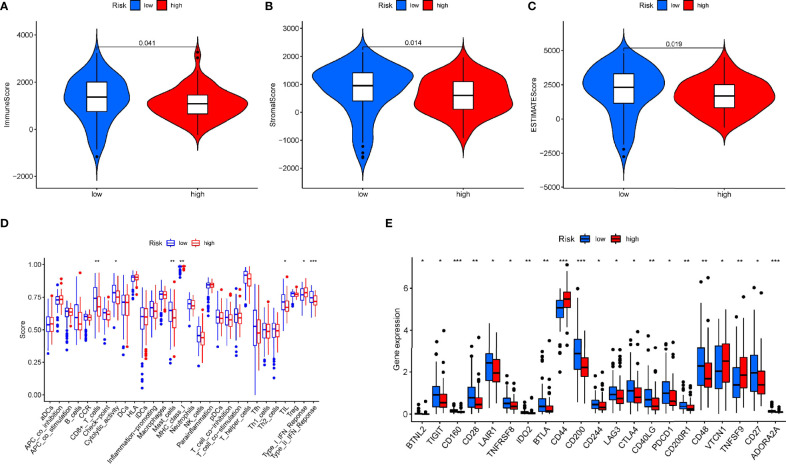
The relationship of the prognostic signature and immune cell infiltration in PAAD. **(A-C)** The immune score and stromal score in high and low risk group. **(D)** Comparison of the enrichment scores for immune cells and immune-related pathways between low- and high-risk group. **(E)** The differential expression of immune checkpoint between high-risk (red box) and low-risk (blue box) group in PAAD. *p < 0.05; **p < 0.01; ***p < 0.001.

**Figure 8:**
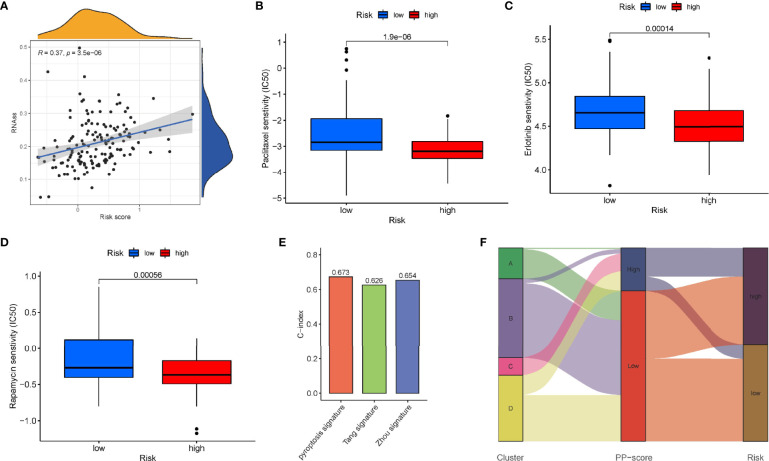
Sensitivity to chemotherapeutic agents in the high - and low-risk groups. **(A)** RNAss between high- and low-risk groups. IC50 of **(B)** paclitaxel, **(C)** erlotinib and **(D)** rapamycin between high- and low-risk groups. **(E)** Comparison of the C-index of our model with other previously established models. **(F)** Sankey diagram showing the correlation among tumour classification, PP-score and risk signature.

**Figure 9:**
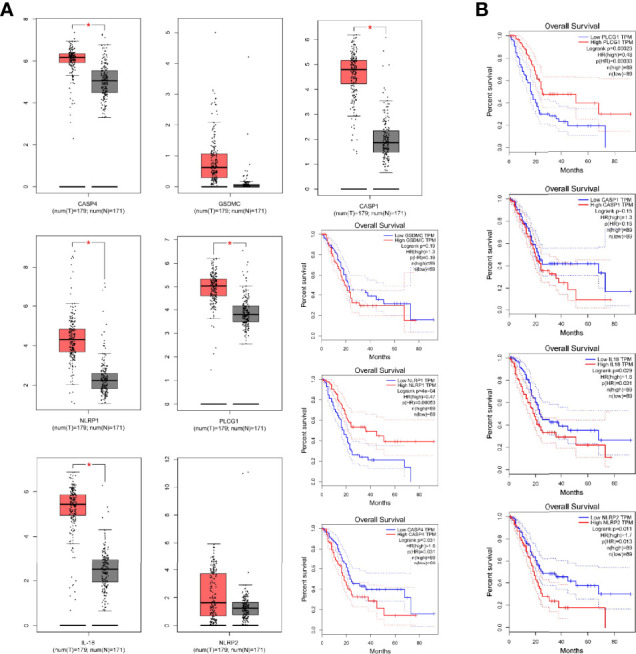
The Differential expression of pyroptosis-related genes in prognostic model. **(A)** The expression of CASP4, NLRP1, PLCG1, GSDMC, IL-18, NLRP2 and CASP1 by GEPIA. **(B)** Kaplan-Meier survival analysis for above PRGs. *p < 0.05.

Immune scores and stromal scores calculated according to the ESTIMATE algorithm showed that low-risk patients had higher immune and stromal scores, which suggested that low-risk patients had a lower tumor purity (**Figures 7A–C**). In addition, single-sample geneset enrichment analysis (ssGSEA) was performed for quantifying immune cells and other immune functions, which showed that CD8^+^ T cell was greatly improved in the low-risk group (**Figure 7D**). Further, we investigated the differential expression of multiple immune checkpoints between high and low risk patients. We found that the expression level of each immune checkpoint gradually improved in the low-risk patients, including CTLA4 and PD-1, which indicted that low-risk patients are more likely to benefit from immune checkpoint therapy (**Figure 7E**). Meanwhile, the m6A expression level in high-risk and low-risk patients was also analyzed. Most m6A-related genes were remarkable increased in the low-risk group, including YTHDC2, METTL14, ALKBH5, METTL3 and WTAP, only the expression of HNRNPC was decreased (**Supplementary Figure 4**). The result demonstrated pyroptosis may be related to m6A modification. Furthermore, the RNAss between high and low risk patients were analyzed, which showed that higher risk was associated with higher RNAss (**Figure 8A**). We also analyzed the sensitivity of high- and low-risk patients to chemotherapeutic agents. The result showed that patients in the high-risk group were more likely to benefit from chemotherapy, including rapamycin, paclitaxel and erlotinib (**Figures 8B–D**). Further, we compared our model with other previously developed models (18, 19), and the C-index displayed that the model we built had more predictive value (**Figure 8E**). What’s more, as shown in **Figure 8F**, all patients in cluster C were classified into high PP-score, which was accompanied by a worst survival outcome. Moreover, we evaluated the expression profiles of these seven PRGs in prognostic model. GEPIA results showed that CASP4, NLRP1, PLCG1, IL-18 and CASP1 were significantly upregulated in PC than in normal tissue, which indicted that these PRGs may involve in the tumorigenesis of PC (**Figure 9A**). In addition, the Kaplan-Meier survival analysis indicted the prognostic ability of these seven PRGs (**Figure 9B**).

### Validation of PRGs in Prognostic Signature

**Figure 10:**
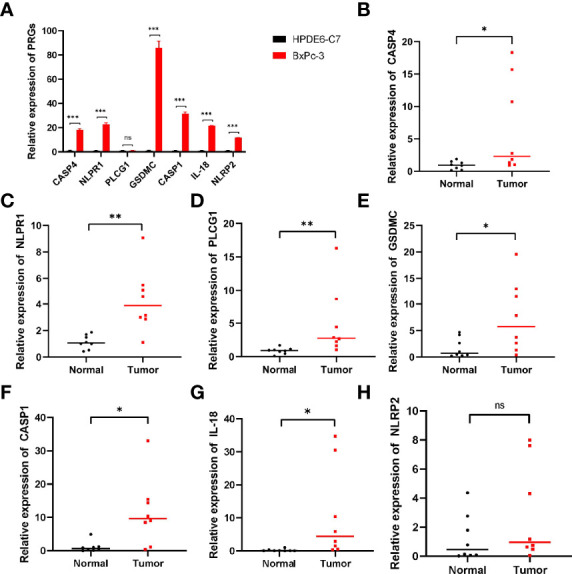
qRT‐PCR showing the expression of prognostic PRGs in cells and tissues. **(A)** The expression of pyroptosis-related genes in PC cells. **(B–H)** The expression of PRGs in prognostic model in PC tissues. ns not significant; *p < 0.05; **p < 0.01; ***p < 0.001.

qRT-PCR revealed the relative expression of CASP4, NLRP1, PLCG1, GSDMC, IL-18, NLRP2 and CASP1 in PC cells and tissues. As shown in **Figure 10A**, all these genes were significantly upregulated in PC cells compared with normal groups except for PLCG1, which had no difference in cell. In PC tissues, only NLRP2 expression was not significantly different **(Figures 10B–H)**. These results suggested that these prognostic PRGs may involve in the tumorigenesis of PC.

## Discussion

Pancreatic cancer is an abdominal malignant tumor with high mortality, and the the difficulty of early diagnosis and the lack of effective treatment lead to poor prognosis (3). As a consequence of the lack of specific clinical manifestations for PC patients in the early stage of the disease, most of the patients have reached the middle and late stage of diagnosis, and are no longer eligible for surgical resection (20). The incidence of pancreatic cancer is on the rise over recent years, and its mortality rate ranks third among malignant tumors (21). Thus, there is an urgent need to study effective molecular biomarkers to establish a theoretical basis for the diagnosis and clinical treatment of PC patients.

Pyroptosis is an inflammatory cell death triggered by a variety of pathological factors such as infection, malignant tumor, etc (22). Pyroptosis is mediated by caspase family proteins and characterized by rapid rupture of the cell membrane and the release of intracellular pro-inflammatory substances (23). It has been reported that there are three pathways for pyroptosis, including a classical one that depends on caspase-1, a non-classical one that depends on caspase-4/5/11, and a special one that depends on caspase-3 (12). Studies have shown that GSDMD and GSDME are critical molecules in pyroptosis (24, 25). With the deepening of research, the relationship between pyroptosis and tumor therapy has been widely concerned. Pyroptosis can promote the growth of malignant tumor through the inflammatory microenvironment formed by proinflammatory action or affecting some signaling pathways (26). However, many studies have confirmed the anti-malignant effect of pyroptosis, which can play a key role in the treatment of malignant tumors by regulating some targets or signaling pathways (27, 28). It has been shown that down-regulation of GSDMD can significantly promote the proliferation of gastric cancer *in vitro* and *in vivo* (29). In addition, NLRP3 can be used as an important prognostic marker for nasopharyngeal carcinoma patients (30). Inhibitory effect of pyroptosis on pancreatic cancer has also been reported. Overexpression of MST1 promotes pyroptosis by increasing the level of ROS (31), thus inhibiting the proliferation, migration and invasion of PDAC.

In our research, we screened 33 pyroptosis-related genes (PRGs) from previous literatures, and expression matrix of these genes was extracted from the TCGA database. Subsequently, univariate COX analysis was used to screen for PRGs associated with prognosis, which were the basis for subsequent analysis. Firstly, we performed a consistent cluster analysis for PAAD pantients based on prognostic PRGs. We found that the survival in cluster B was higher than that of other clusters. Further, in order to more accurately evaluate the prognosis of individual tumor patients, a set of scoring system was established to quantify the pyroptosis level base on these pyroptosis-related genes. As we can see from the scoring system, patients with low PP-score had a prolonged survival compared to high PP-score. At the same time, we observed that patients in cluster C were significantly associated with higher PP-score, and both of them had a poor prognosis. Finally, to evaluate the risk and prognosis in pancreatic cancer more conveniently using pyroptosis-related genes, we constructed a prognostic model consisting of seven PRGs, including CASP4, GSDMC, NLRP1, PLCG1, IL-18, CASP1 and NLRP2. Hou et al. (32) discovered that GSDMC was specifically cleaved by caspase-8, thereby transforming apoptosis into pyroptosis and promoting tumor necrosis. Studies have shown that PLCG1 is involved in GSDMD-N-mediated pyroptosis in a calcium-dependent manner (33). In recent years, immunotherapy has been used to treat many types of tumors, but the clinical trials in pancreatic cancer have not achieved the expected effect (34). One of the main reasons for the failure of immunotherapy is that the tumor microenvironment in pancreatic cancer is immunosuppressive (35). Therefore, immunosuppressive cells and molecules in pancreatic cancer provided potential targets for immunotherapy. Therefore, we quantified immune cells and immune functions between high-risk and low-risk group, and found that CD8+ T cell was greatly improved in the low-risk group. In addition, both immune and stroma scores were higher in the low-risk group, suggesting lower tumor purity in the low-risk group. Studies have shown that increased PD-L1 expression predicts poor prognosis in pancreatic cancer (36, 37). We also analyzed the correlation between the risk score and expression of multiple immune checkpoints, and confirmed that the expression level of each immune checkpoint gradually improved in the low-risk patients, including CTLA4 and PD-1, supporting the possibility that the low-risk group would benefit more from immune checkpoint therapy.

## Conclusion

In summary, we used consensus clustering to distinguish PC patients in TCGA into four subtypes, among which there were significant differences in survival. Notably, to quantify the pyroptosis level of individual tumor, pyroptosis score (PP-score) was constructed to evaluate the pyroptosis pattern of individual PC patients. Moreover, we developed a prognostic risk model that used pyroptosis-related genes to reflect the pyroptosis risk. We expect that our research could provide a new perspective on the carcinogenic mechanism and potential targets of pancreatic cancer treatment. But admittedly, Our research still has some limitations. All analyzes were performed in public databases, so it is recommended to validate in combination with our own sequencing data. In addition, *in vivo* and *in vitro* experiments need to be performed to further confirm our results.

## Data Availability Statement

The original contributions presented in the study are included in the article/**Supplementary Material**. Further inquiries can be directed to the corresponding author.

## Ethics Statement

The studies involving human participants were reviewed and approved by the Ethics Committee of PLA General Hospital. The patients/participants provided their written informed consent to participate in this study.

## Author Contributions

LL, ZD, and ZX made the same contribution to this study and were recognized as co-first authors. LL was involved in the conception of this study and wrote most of the manuscript. ZD was responsible for downloading the corresponding data from public databases. ZX processed and analyzed the obtained data. WZ participated in writing parts of the manuscript. RL polished the manuscript. All authors contributed to the article and approved the submitted version.

## Conflict of Interest

The authors declare that the research was conducted in the absence of any commercial or financial relationships that could be construed as a potential conflict of interest.

## Publisher’s Note

All claims expressed in this article are solely those of the authors and do not necessarily represent those of their affiliated organizations, or those of the publisher, the editors and the reviewers. Any product that may be evaluated in this article, or claim that may be made by its manufacturer, is not guaranteed or endorsed by the publisher.
