# Supplementary material for: Analysis of Pyroptosis-Related Signature for Predicting Prognosis and Tumor Immune Microenvironment in Pancreatic Cancer
Source: Front Oncol. 2022 May 31;12:770005. doi: 10.3389/fonc.2022.770005 (PMC9192978; doi:10.3389/fonc.2022.770005)
Supplement: Supplementary file 1 [file DataSheet_1.docx]

Supplementary Material


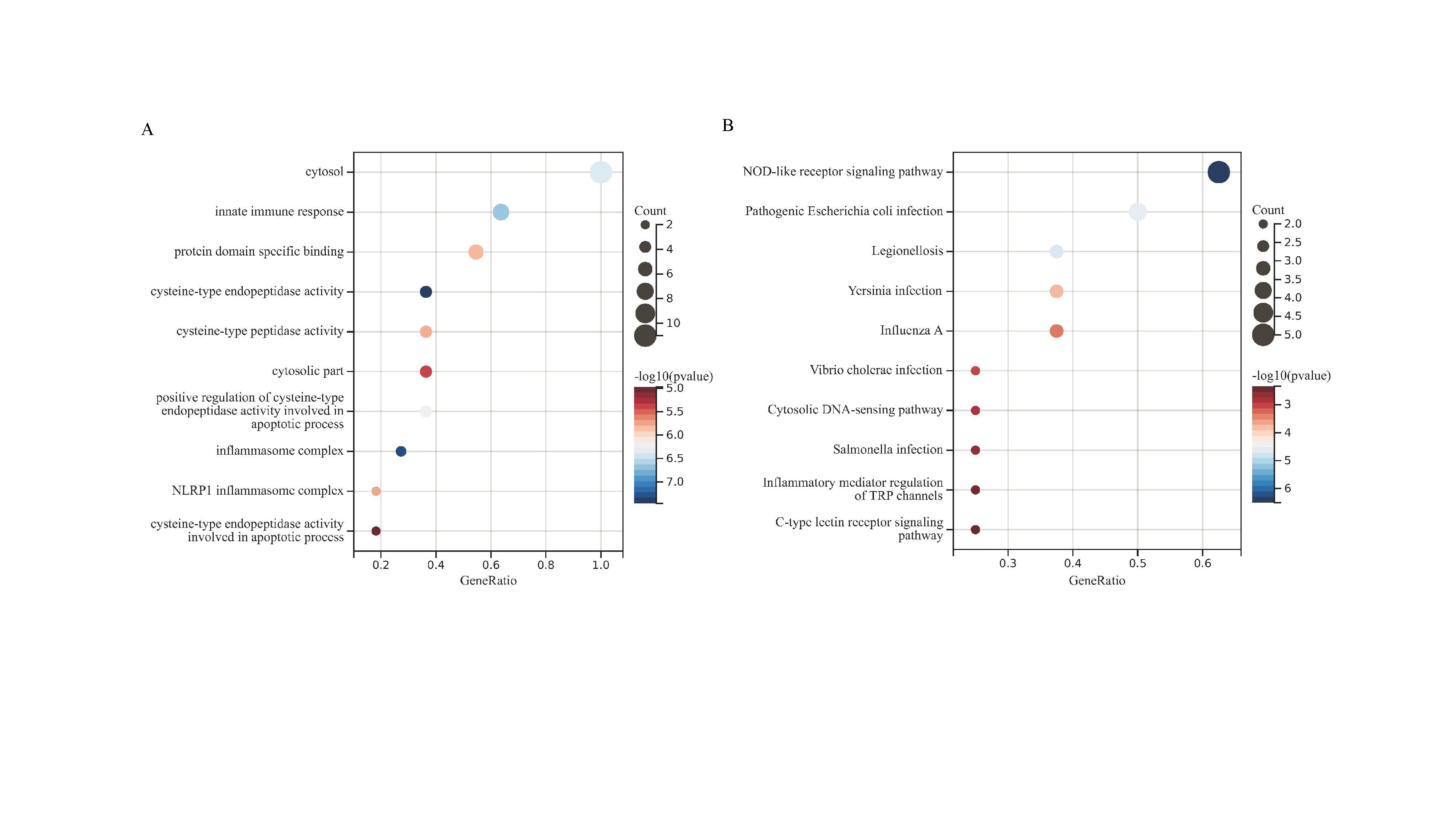


**Supplementary Figure 1** Functional analysis based on the prognostic PRGs. **(A)** Bubble graph for GO enrichment and **(B)** KEGG pathways (the bigger bubble means the more genes enriched).


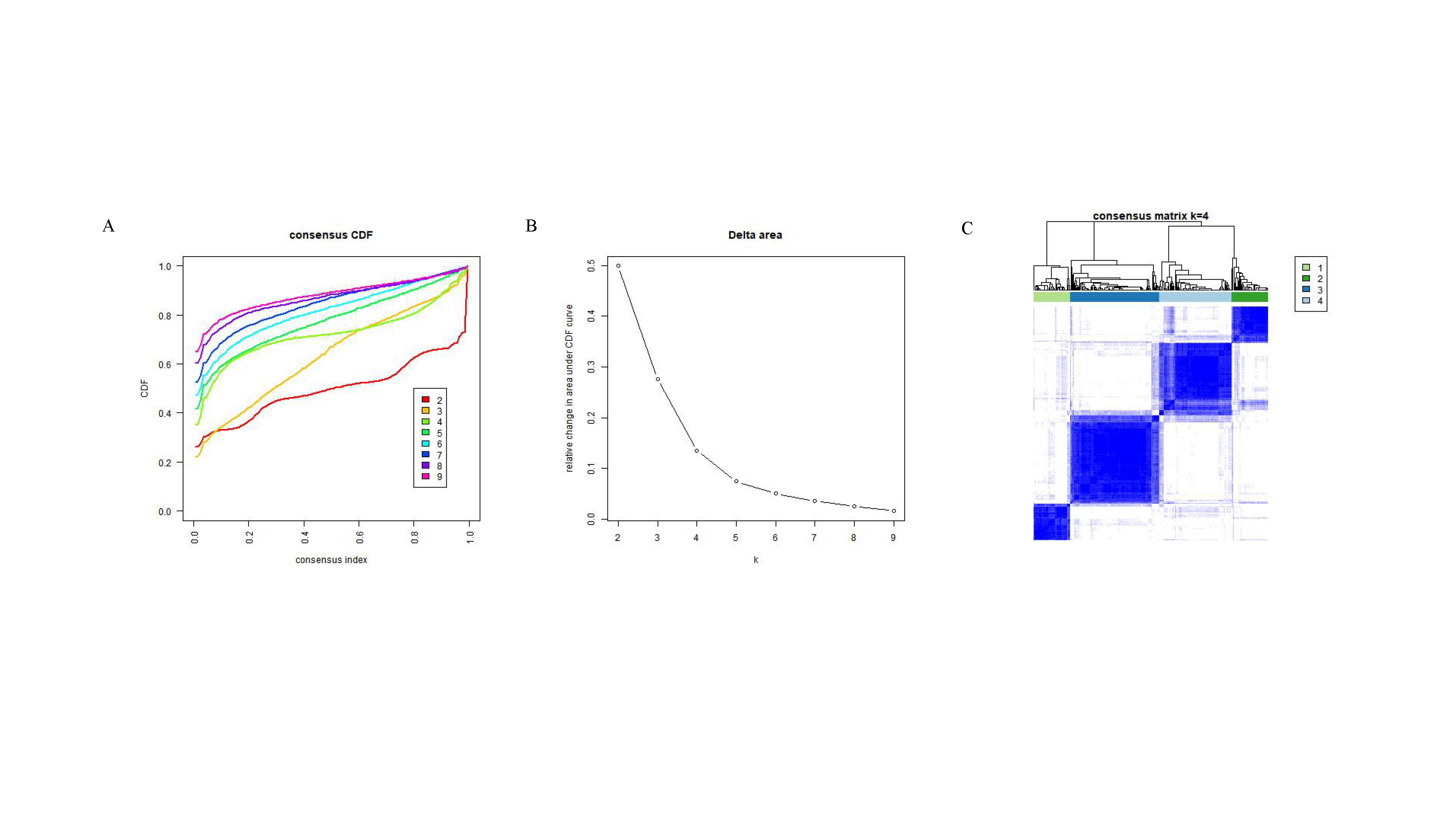


**Supplementary Figure 2** Tumour classification based on the prognostic PRGs in E-MTAB-6134.


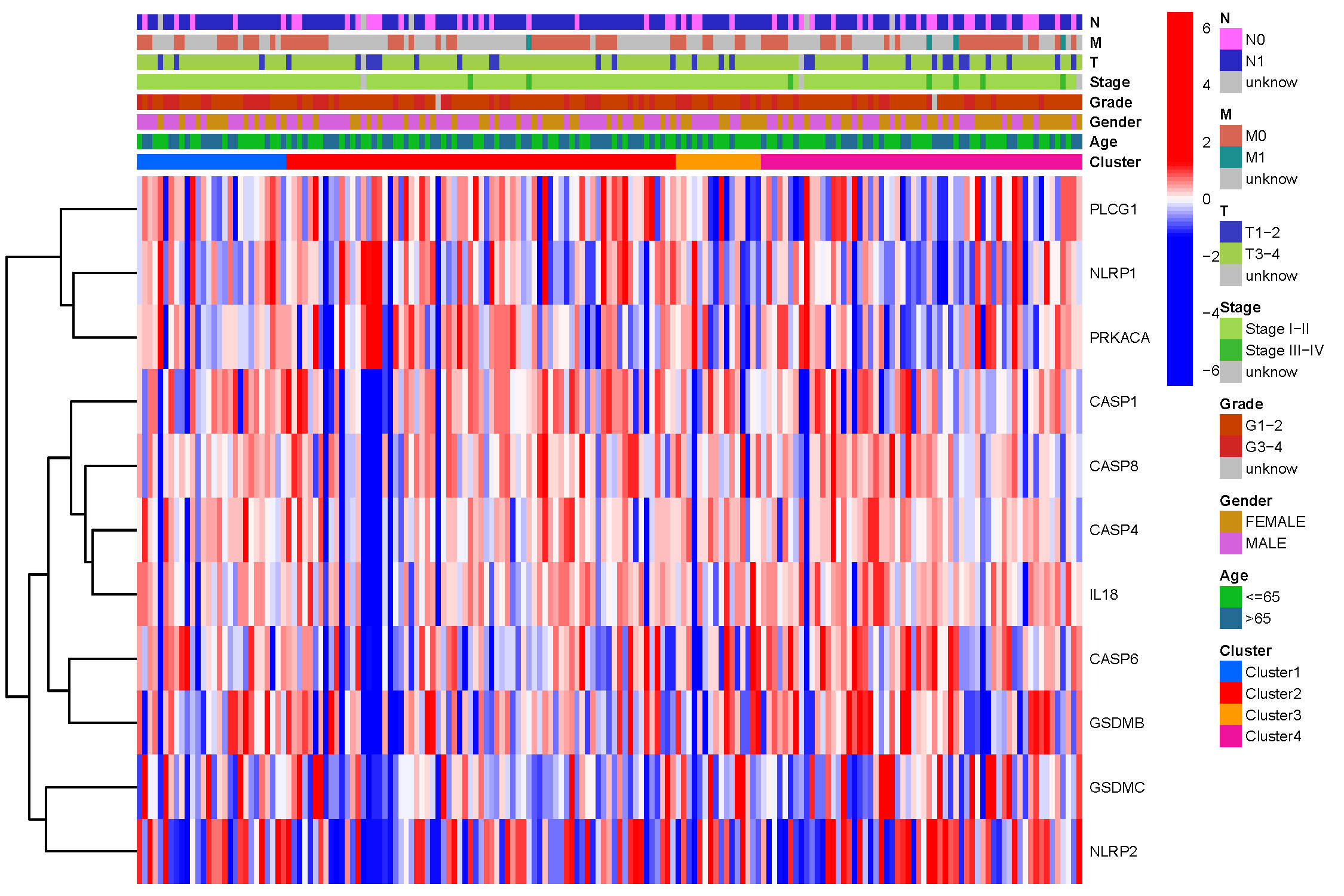


**Supplementary Figure 3** Heatmap between tumour classification and clinicopathologic characteristic of PC patients.


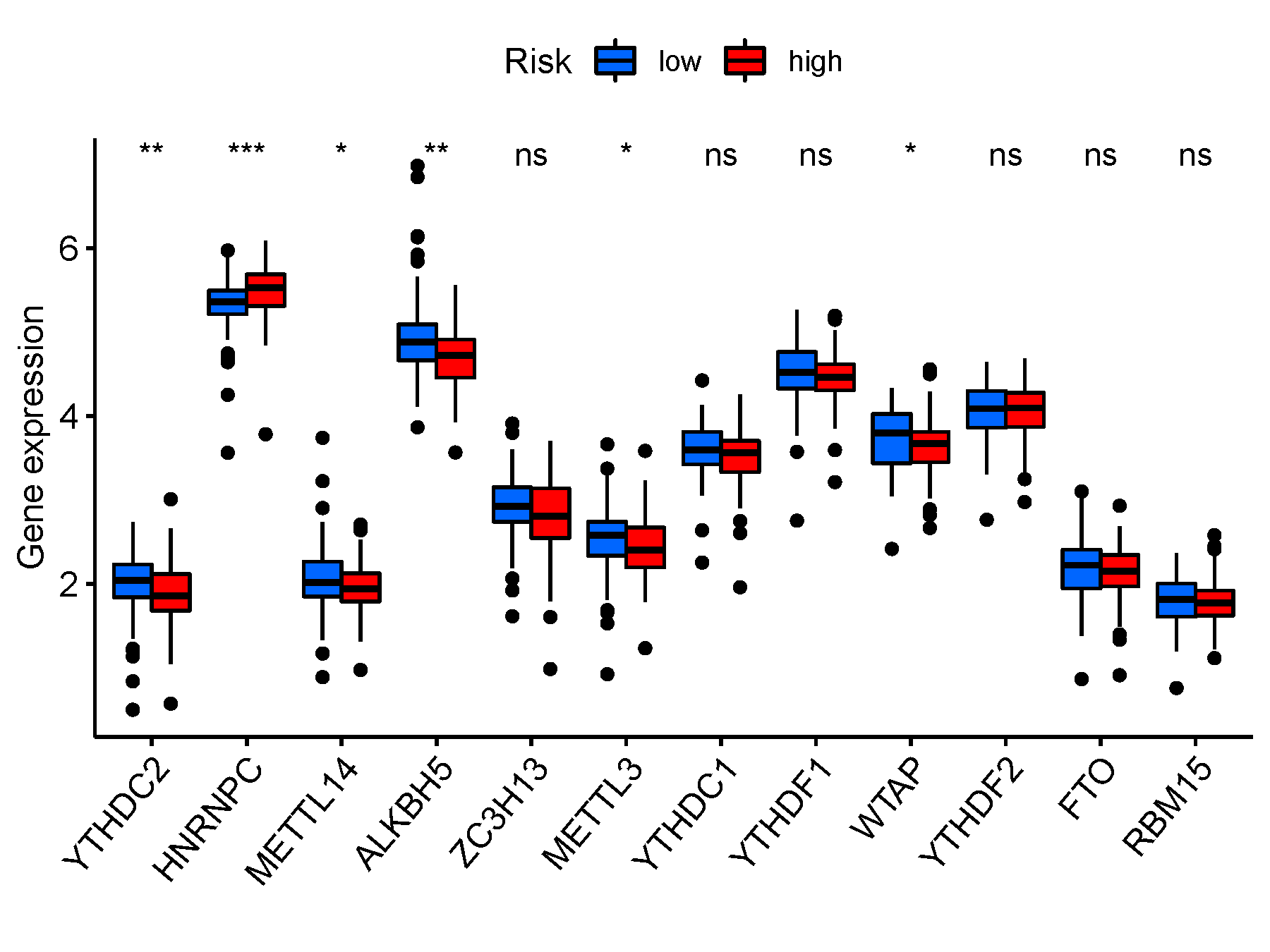


**Supplementary Figure 4** The differential expression of m6A-related genes between high-risk (red box) and low-risk (blue box) group in PAAD. *p < 0.05; **p < 0.01;***p < 0.001; ns, not significant.

.
